# Value-based requirements engineering: method and experience

**DOI:** 10.1007/s00766-017-0273-y

**Published:** 2017-06-06

**Authors:** Sarah Thew, Alistair Sutcliffe

**Affiliations:** 1 Greater Manchester Academic Health Sciences Network, Manchester, UK; 2 Manchester Business School, University of Manchester, Manchester, UK

**Keywords:** Requirements elicitation, Values, Motivations, Emotions

## Abstract

‘Socio-political’ issues, such as emotions, values and people’s feelings, are often cited as problems in the RE process. A method is described for analysing such issues. The method consists of a taxonomy of stakeholders’ values, motivations and emotions (VME), with process guidance for eliciting and analysing these issues for the RE process and design implications. Values are personal attitudes or long-term beliefs which may influence stakeholder functional and non-functional requirements. Motivations are psychological constructs related to personality traits which may be viewed as stakeholders’ long-term goals in RE. Emotions are cues to stakeholders’ reactions arising from value/motivation conflicts. The method is supported by a website which illustrates the taxonomy with explanations and scenarios describing problems arising from value conflicts, and from poor understanding of stakeholder values. Two method validation studies were undertaken: first, an evaluation of the website and method by novices and RE experts; and second, case study applications of RE value analysis in real-world industrial practice. The method was used by all practitioners, although in different ways, some used it to create an agenda of issues for analysis while others employed the VMEs to interpret stakeholders’ views and manage stakeholder negotiations. The validation studies provide evidence for the acceptability of the method for industrial practitioners, illustrating how value-related problems are identified and analysed effectively by the method. The utility of analysing VMEs is compared to other ‘socio-political issues’-oriented methods in RE and methods which focus on monetized values in product requirements.

## Introduction

Socio-political issues are often cited as problems in the RE process [1–3], although there is little advice about how to deal with these issues. Few studies have directly considered stakeholders’ emotions during the analysis phase, although the impact of negative stakeholder emotions is known to impair system acceptance and use after implementation [2, 4, 5]. Gowler [6] observed that systems must fit with stakeholders’ values and beliefs to be successful. However, it is not easy to gain insight into stakeholder values or emotions, since people rarely directly express such information. In this paper, we attempt to make such knowledge explicit and propose a method for analysis of socio-political issues in RE.

Value in RE has been analysed in the sense of worth or monetary value, notably in the *e*-value method which had been applied to business process analysis and socio-technical systems requirements [7–9]. However, values may also be interpreted as a set of issues which are frequently referred to as problematic in the RE process, such as politics, culture, sensitivities about the consequences of automation and conflicts between stakeholders [4, 10]. While some socio-political issues such as responsibility and authority, are modelled in i* [11] and extensions thereof [12], stakeholder values, motivations and emotions (VME) have not been explicitly included in requirements processes to date. Non-functional requirements are related to values when they describe qualities such as accuracy, privacy or security; hence, one motivation for this work is to explore the antecedents of non-functional requirements in the language stakeholders employ when describing their domain.

In this paper, we describe the development, validation and deployment in industry of a method for value-based requirements engineering which guides the elicitation of stakeholders’ values and motivations underlying socio-political issues in systems development as well as taking stakeholders’ potential emotional reactions to system change into account. The main research question we address is methodological: “can an effective method for value-based requirements engineering be specified?” and the consequent validation question “can the value-based RE method be effectively used by industrial practitioners?”. Implicitly, we also investigate adaptation and adoption of RE methods in practice. This paper builds on Thew’s thesis research [34] and extends our previous work on user-centred RE [50, 55] by reporting an extended version of the VME taxonomy in [55] as well as describing several validation case studies on the value-based requirements analysis method. The following sections of this paper first cover related work and then describe the background for the value-based requirements engineering (VBRE) method and the value taxonomies. Then the method process and website support tool are described. This is followed by three case studies into method use, the first two report application of the method in two e-science projects, while the third reports a more extensive study into industrial deployment with four analysts. The paper concludes with a discussion comparing VBRE with related methods and reflections on RE methodology.

### Background and related work

Appropriate consideration of social and political issues has been recognised as key aspects of successful requirements engineering for some time [13, 14]. Such issues often involve stakeholder values which might be compromised by a system design [5, 23]. Values which may be considered as stable, personal beliefs, [38] are closely related to motivations [43] which also influence stakeholders’ views about a system, while motivations may be viewed as stakeholders’ long-term goals [21]. Conflicts between stakeholders’ values and motivations and solutions proposed by requirements analysts may be tacit, and hence awareness of these issues and means of eliciting them are important. One cue to value and motivation clashes can lie in stakeholders’ emotional reactions [48]. Sensitivity to emotional reactions is therefore one means of eliciting potential value-based problems, and hence the method we propose addresses VMEs, values, motivations and emotions. Values and motivations form inputs to the requirements specification process, while emotional awareness we argue is a valuable analytic tool. A review of power and political issues in RE [15] pointed out that requirements are socially constructed in a political context and advocated development of techniques for social power modelling in RE. Stakeholder conflicts and their socio-political causes have been analysed as critical RE issues [2, 3], although most studies tend to be post hoc analyses of system failure rather than recommendations about how to address such issues a priori. Stakeholder conflicts often arise from stakeholders’ values and emotional reactions which have been noted as important influences in socio-technical RE where adverse emotional responses can lead to system rejection [4, 5]; for example, stakeholder values of ownership and control can lead to frustration and rejection of ERP systems [5].

A variety of methods aiming to support requirements analysts in the identification of socio-political issues have been published: the use of discourse analysis [16–18], Ramos and Berry’s guidelines [4] and the application of ethnography to the requirements elicitation process [19]. User values have been analysed at a high level of cultural attributes such a power distance and individualism [20], while Sutcliffe et al. [21] argued that cultural values should have an important influence on requirements definition; however, apart from some examples in specific applications [e.g. 22], few reports of application of value-based requirements analysis have emerged. In human–computer interaction, the value-based design method [23] provides a process for eliciting stakeholders’ feelings and attitudes to potential systems by presenting cue cards associated with possible emotional responses and user values. Scenarios and storyboarding techniques are used to elicit stakeholder responses, but value-based design does not focus directly on requirements definition; instead, it aims to elicit stakeholders’ attitudes and feelings about products and prototypes as an aid towards refining requirements with human-centred values. No specific guidelines were given for identifying the elicited values or their implications. Values and affective responses have been investigated by Cockton et al. [24] in worth maps, which attempt to document stakeholders’ views about products or prototypes. Worth maps may include values and emotional responses, but their main focus, similar to value-based design, is to elicit informal descriptions of potential products expressed in stakeholders’ language of feelings, values and attitudes.

RE methods for analysing values in the sense of economic worth for services [7] and for anticipating overall customer value in product lines by applying a road-mapping process in strategic product release planning [25] have been proposed. However, only general advice about considering political and social issues is given in most RE methods [1, 26]. A more detailed taxonomy of social and political RE issues with guidelines for recognising affective reactions among stakeholders was proposed by [4] who applied their approach in analysing requirements for ERP applications. In an application of Activity Theory to RE, Fuentes-Fernandez et al. [27] elaborate UML schemata for social issues and propose patterns for recognising stakeholder conflicts; however, they do not give specific advice about eliciting or analysing stakeholders’ values and emotions. In conclusion, although socio-political issues have received considerable attention in ethnographic studies for RE [14, 16], and the importance of understanding socio-political issues has been highlighted in general RE methods [1, 13], this research has not produced specific guidance about how to analyse stakeholders’ values and related concepts. Mumford’s ETHICS method [17] is relevant to our research, since it provides detailed guidance on identifying values, using a taxonomy and a questionnaire-based approach to support analysts in identifying stakeholders’ values. However, this method does not provide clear guidance to analysts on the identification of stakeholders’ values.

Values appear to be tacit knowledge: we recognise them when we encounter them but trying to articulate them beforehand is difficult. As noted by researchers in ethnography, such knowledge is contextualised and can only be understood by reference to a situation [19, 28]. The key to understanding values is through language with reference to existing knowledge held by the individual [29]. Questionnaires have been the technique of choice for psychologists when investigating values [30] covering specific topics such as commitment to employment, personal development, professionalism. The Models of Man questionnaire has been used to assess the impact of systems change within organisations [10, 17] and covers the personal background of respondents, their perceptions of different groups involved in the systems design process, their objectives, and criteria for job satisfaction and personal values. However, questionnaires are usually framed at a specific level which hinders their application to general requirements analysis.

Unlike values and motivations, emotions are reactive responses to events, objects and artefacts. Software developments have the potential to change working circumstances and therefore to have an emotional effect; for example, a system which changes the power balance in a relationship, or information previously ‘guarded’ by a stakeholder becoming widely available, could raise anxiety. Investigations into the elicitation of emotions have observed that people rarely speak directly about them; rather, they illustrate their emotions through stories or speech patterns [31, 32]. Creating narratives and scenarios facilitates understanding our experiences and is a natural way to share experiences with others [33]. There are few examples of elicitation techniques designed to identify emotions although the Underlying Discourse Unveiling Method does provide some guidelines [19]. Ramos et al. [5] note the shortage of guidelines to help elicit emotions, beliefs and values from the visible and shared constructions of human action and interaction.

## Method development

### Current analysis practice

A series of interviews with 12 industry analysts explored their views on the relevance of stakeholders’ VME, the impact on their RE work, and the adoption of techniques or strategies to aid the analyst in identifying such information. A brief summary of the findings is given in this paper; for further details see [34].

Differences between novice and experienced analysts were apparent, both in the appreciation of the impact of VMEs and in the explicit use of techniques to identify this knowledge. The investigation identified behaviours adopted by expert analysts that would be useful to promote in novice analysts and documented the analysts’ own requirements for a method to support them in eliciting VME.

Several analysts reported occasions when they had made use of their common sense understanding about stakeholder values or emotions, either in management of the requirements process, or in system design. The majority of analysts considered understanding stakeholders’ values and emotions as a serendipitous benefit that “just happened” when they were particularly perceptive. Novice analysts commented that they felt this area of expertise had been neglected in their training, and three stated that they felt anxious about their lack of knowledge for gathering or acting on this information.

The consensus expert approach was to anticipate what might be important to their stakeholders, and their likely emotional response to the project; followed by observation or exploration of stakeholders’ values and feelings as ‘hunches’ to guide the requirements elicitation and analysis process. It appears that analysts do recognise the usefulness of an awareness of stakeholders’ values and emotions for improving their management of the requirements process as well as for anticipating social sensitivities in system requirements; however, the majority of analysts see this understanding as largely serendipitous, while only a minority articulated elicitation techniques they had developed through experience. The interviews revealed a widespread concern over the need for better awareness of the impact of socio-political issues in RE and that there was no guidance for handling such issues. Some analysts commented that dealing with socio-political issues was a matter of experience. The consensus opinion was that better guidance was needed, and that current practice varied according to experience and individual ability. Some analysts had better emotional intelligence but most felt that training and practice in the area should be improved. The findings from the interviews with analysts indicate that more experienced analysts have an increased awareness of stakeholders’ VME. In contrast, novice analysts commented that it would not usually occur to them to think about stakeholders’ VME, until there were problems in their project, while expert analysts described taking time to reflect on stakeholders’ VME. In similar studies in the literature, major differences in problem identification skills and political and social awareness between novice and expert analysts have been reported [35, 36], as have the need for improved analyst communication skills and the importance of rapport building [1, 26].

This suggests that novice analysts could benefit from adopting some of the behaviours described by the expert analysts, periodically reflecting on the VME expressed by stakeholders, and asking questions to explore these aspects of the their work and attitudes towards the project. The analyst interviews also identified requirements that any method aiming to support analysts in working with VME would need to address in order to be acceptable and of practical value:

- Minimal additional demands on time: analysts generally have limited time with stakeholders and are usually busy themselves; therefore to have any chance of being adopted by analysts, any technique should place as few demands on their time as possible.
- Culturally acceptable to analysts: a number of the analysts interviewed in [34] did not wish to directly question stakeholders’ about their VME. Reasons for not doing so included belief that direct questioning would be inappropriate, and discomfort at asking questions about socio-political issues when they perceived themselves to be working within a technical role. Furthermore, while there is good evidence to show that stakeholders’ VME can have a significant impact on the outcomes of a project, investigations into such information is not traditionally part of the remit of a software development project. Therefore, consideration also needs to be given to the nature of an investigation into stakeholder VME for both the analyst and the stakeholder. When carrying out academic research, both the researcher and the participants in a study are generally not concerned about the long-term consequences of such a study. In contrast, both requirements analysts and their stakeholders are concerned with obtaining their preferred outcomes for a project, and usually with maintaining some longer-term relationship such as further project work or software support. Attempts to encourage analysts to consider such information should be sensitive to the ongoing nature of the analyst and stakeholder relationship.
- Should not require any special expertise or skills beyond those that could be expected of a requirements analyst. These include the ability to develop effective relationships with stakeholders, elicit and communicate requirements and consensus building.
- Should build on and/or integrate with existing established and commonly used RE practices such as interviews, observation, workshops, iterative design [1, 13, 26].

Furthermore, any approach to examining stakeholders’ VME ‘in the wild’ needs to be open-ended and context sensitive, since the circumstances of every project and every stakeholder will be different. Analysts need support in thinking about and looking for evidence of VME in the particular context of their own projects, but may also need prompting about potential VME they might wish to consider; therefore, any approach should not prescribe particular courses of action or conclusions but rather encourage analysts to determine what is appropriate for their own project, considering alternate explanations and challenging initial assumptions.

### VBRE taxonomies

A literature survey was conducted to elicit psychological definitions of values and related phenomena. Definitions of value vary from worth and desirability, to judgement of what is valuable or important in life (OED). In psychology, values are beliefs and attitudes held by people about other people, organisations or artefacts; for instance, in Small Group Theory [37], values, beliefs and attitudes are held by group members and influence the group operation, collaboration and performance. Kluckhohn’s [38] definition gives a faceted classification, including modality (positive or negative), intensity (of belief), content (with a very limited subtaxonomy of aesthetic, cognitive or moral), generality (of application), extent (belief in a population), and explicitness (in articulation). This schema has been adopted by other studies into values in general [39], and in the context of software development [10, 17]. Rescher’s value theory [39] provides a useful classification of the objects to which values may be applied: things, the environment, people, groups and whole societies; while another facet classifies the potential benefit of applying values such as economic, moral, social, political, aesthetic, and religious. However, some classes are difficult to interpret, such as professional, sentimental, or intellectual benefit.

The taxonomy of values and their consequences for process guidance are illustrated in Table 1. Eight upper-level value categories are proposed based on Rescher’s theory [39] and our own investigations from card-sort experiments and expert interviews. Six categories accord with generally recognised concepts, some of which have more stable interpretations: trust, morals, aesthetics and security; while sociability and creativity/innovation hide many subcategories. Some of these are given in the related terms column. Personal characteristics are also diverse, so personality theory dimensions (introvert/extrovert, sensing/intuition, thinking/feeling, judging/perceiving) are used, with some additions. Unlike other values, personal characteristics are simpler attributes which describe people. Personality characteristics are closely related to motivations and both have implications for team management in the RE process and customisation for personal RE [21, 40]. Motivations are a placeholder for a more detailed taxonomy (see below), while beliefs and attitudes are a diverse category including socio-political, cultural, and religious beliefs. These change more rapidly than other value clusters which are more closely related to personal attributes; consequently, we have not elaborated this part of the taxonomy. Related terms were derived from card-sorting studies described in [34].

**Table 1:** Values: elicitation hints and implications for RE process management; see also [55]

Value concept	Related terms	Potential sources	Process implications
Trust	Openness, integrity, loyalty, responsibility reliability	Relationships with other individuals/departments Privacy policies	Less control milestone checks improved team confidence
Collaboration	Cooperation, friendship, sympathy, altruism	Relationships with others Awareness of others (office politics)	Improved team cooperation shared awareness
Morals/ethics	Justice, fairness equality, tolerance	Behaviour towards others Opinions of others’ behaviours	Openness and honesty in team
Creativity innovation	Originality, adventure, novelty	Work processes, problem solving	Creativity workshops facilitators
Aesthetics	Beauty, nature, art	Self-appearance Reaction to images, shapes, art and design	Team members designers storyboards
Security	Safety, privacy, risk	Data management policies, Attitudes towards change	Hazard/threat analysis
Personal characteristics	Serious/playful, introvert/extrovert Systematic/opportunistic	Self-image Personae scenarios Psychological questionnaires	Customisation analysis for personal RE. Team conflict management
Motivation	Ambition, achievement *see also* Table 2	Ambitions, goals, career plans	Stakeholder analysis, rewards, incentives for members
Beliefs and Attitudes	Cultural, political, religious topics	Leisure interests Stakeholder background Reaction to news events	Stakeholder analysis team composition, incentives

The elicitation guides in column three suggest some potential conversation topics which might expose particular values.

The process implications in column 4 vary from organising the team composition in response to aesthetic needs (i.e. include aesthetically aware designers), specialisation of the RE process to include safety and risk analysis [41, 42], to more general heuristics for project team management, such as the need for fewer controls when trust is high, or the converse when mistrust is discovered. Sensitivity to moral values indicates the need for honesty, openness and fairness in all parts of the development process. In many cases, especially with motivations and attitudes, value analysis may alert the analyst to potential stakeholder conflicts, when negotiation will be necessary to arrive at a common set of values. Alternatively, system configuration/customisation may need to be considered (e.g. different levels of security controls mapped to stakeholders who regard security as very or not important).

When most values are not found, then the advice is not necessary, but the absence of, or negative values for, trust, sociability or morals may need corrective action to ensure a productive relationship among the project team.

### Motivation analysis

Motivations are related to personality, and can be considered as long-lasting, high-level personal goals [40]. They are important for understanding stakeholder groups and for individual-level requirements when systems can be customised or configured. Motivations which equate to short term, transient goals (i.e. objectives) are not considered in the analysis. Table 2 summarises the more important motivations for requirements analysis, synthesised from Maslow’s motivational theories [43] and other more specialised theories of human needs [e.g. 44]. Column 3 suggests implications for each motivation type; for example, self-efficacy, curiosity and learning point towards the need for extendable systems where the stakeholder can take control and customise or programme additional functionality; furthermore, these individuals may tolerate increased learning burdens inherent in more complex systems.

**Table 2:** Motivations and their consequences [55]

Motivation	Description	Implications
Power	Need to control others, authority, command	Work organisation, responsibility, control, hierarchy
Possession	Desire for material goods, wealth	Resource control, monetary incentives, marketing,
Achievement	Need to design, construct, organise	Goal oriented, project aims
Self-esteem	Need to feel satisfied with oneself	Link personal and project goals, praise personal achievement
Peer-esteem	Need to feel valued by others	Team composition, social feedback and rewards, praise
Self-efficacy	Confidence in own capabilities	Confidence building, training, skill matching
Curiosity, learning	Desire to discover, understand world	Extensible systems, self tutoring
Sociability	Desire to be part of a group	Collaboration in work, organisation
Altruism	Desire to help others	Cooperation in work, organisation

Altruism and sociability suggest people who will collaborate and cooperate with others in group working, both in the delivered system and in the system development process. Motivations of self- and peer-esteem can indicate designing systems to suit individual needs; for instance, in e-commerce, marketing tools can be customised to praise customers [45] and thereby improve their self-esteem (positive well being). An example for fostering peer-esteem is giving praise for contributions within e-communities (see [46]) and broadcasting such praise to the whole stakeholder community.

Although motivation analysis applies primarily to individuals or stakeholder groups, some motivations may also be important as properties of organisations, so value and motivation analysis extends the concept of Weltanschauung or world view in the CATWOE taxonomy used in Soft System Methodology [18].

### Analysis of emotions

Emotions can give useful feedback about reactions to project plans and designs, especially since emotional responses are stronger than ordinary opinions and may therefore indicate potential problems leading to stakeholder dissatisfaction or system rejection.

Emotions can be detected via body language, voice tonality and facial expressions [47]. Strong emotions are hard to miss (e.g. anger), but others are less obvious, e.g. a frown could indicate frustration, anxiety or just being puzzled.

The more important emotions and their consequences are given in Table 3. These emotions are based on the classification of emotions as responses (positive or negative) to events, people or artefacts by [48], who list 22 separate emotions. Pleasure, joy and happiness are all positive responses, so no remedial action is needed (see column 3 in Table 3). Fear is usually an overt response, but it may be tacit, for instance, when a new system threatens someone’s job security. Tacit fear may be manifest in a lack of cooperation, non-committal replies, missing meetings and avoiding eye contact during meetings. Anxiety is a milder form of this emotion, probably caused by uncertainty, unwillingness to change and fear of the consequences of a new system on a person’s job, self-esteem and self-efficacy. Frustration may lead to the stronger emotion of anger expressed overtly; more often frustration is not so obvious. The causes may be lack of involvement in the requirements analysis process, being marginalised at the expense of other stakeholders, or not having one’s contributions or values discussed. Frustration in the long-term will be manifest in stress, leading to illness and absenteeism; in the short term stakeholders may be uncooperative and uncommunicative. Frustration can be related to depression, which is a more extreme response to lack of involvement and being marginalised. Finally, disgust and revulsion are strong emotional responses which are usually explicit. The implications are usually obvious from the stakeholders’ reference to a design feature, and this response may be encountered more frequently when aesthetic values and self-image/esteem motivations are important.

**Table 3:** Emotional responses and their possible causes [55]

Emotion	Related feelings	Possible causes	Remedial action
Fear	Fright, worry, threat	Design is personally threatening, negative consequences	Review and remove threats
Pleasure	Joy, happiness	Design is rewarding, positive	None; note for future reference
Anxiety	Uncertainty, worry	Specification may be confusing, consequences not clear, little involvement	Explain specification, use scenarios, reassure stakeholders
Frustration	Annoyance, anger	Irreconcilable conflict, barriers, value-interest clashes, values ignored	Revisit stakeholder analysis
Disgust	Revulsion, horror	Design has complete clash with values/culture	Radical design review
Depression	Withdrawn, isolated, alone	Lack of involvement in process, values ignored	Re-engage stakeholders, improve communication and motivation

## Specification of the method

The taxonomies described in Sect. 2 were incorporated into the VBRE method in an iterative process. Section 3.1 reports the first version of the method and preliminary evaluation. Section 3.2 describes the method refinement which built upon evaluation of the initial version, in particular development and evaluation of the website support tool. Section 3.3 summarises the final version of the method which was applied to the case studies reported in Sect. 4.

### Initial version of VBRE

#### Method procedure

**Fig. 1:**
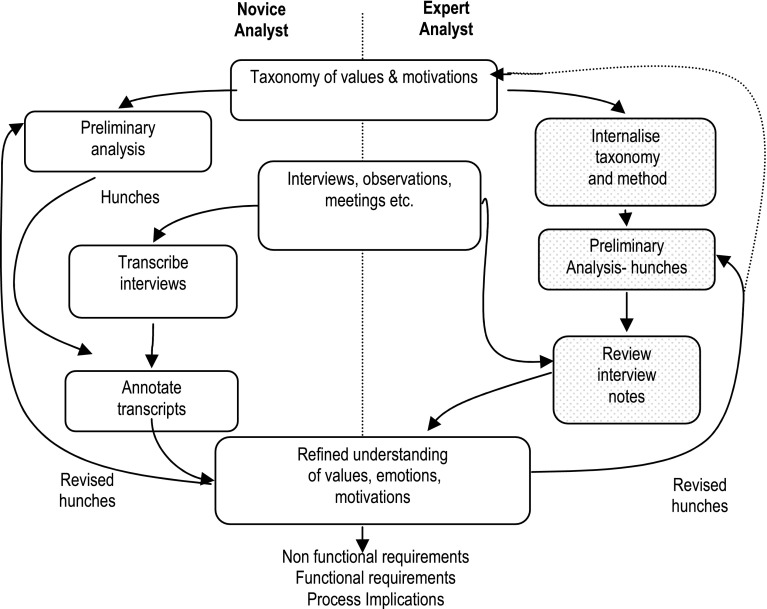
Process stages, and expert and novice pathways in the VBRE method

The key steps of the VBRE method are summarised in Fig. 1. The method can be used in two modes to suit novice or expert analysts and the time resources available. In expert mode, the taxonomy and process are learned in training courses and by experience. The method knowledge is internalised, so it can be used to formulate appropriate questions framed by the analyst’s understanding of the application domain. The method becomes part of the expert’s battery of techniques in scenario analysis, questioning using storyboard and prototype probes; as well as informing review of interview notes, using Tables 1, 2 and 3 as aide memoires.

In novice mode, preliminary analysis of the project circumstances leads to identification of key issues or ‘hunches’, i.e. a subset of the stakeholders’ VME from the taxonomy which seem to be relevant to the application. For example, stakeholders may prioritise certain qualities in their work: creativity, aesthetics and collaboration. Making these intuitions explicit encourages gathering evidence to support or challenge initial hunches.

Interview audio recordings are transcribed to create text records. Annotation involves reviewing transcribed interview notes for evidence of the expression of VME and marking text segments with the taxonomic category, using either the key issues or the tables directly. If time resources are constrained, an alternative is to use expert review of the interview notes stage by listening to audio recordings and making notes.

Experts may also iterate their analysis hence revised hunches are produced as the analysis progress (e.g. when value conflicts emerge). This leads back to preliminary analysis mirroring the loop on the Novice pathway. The tables may be used in conjunction with existing taxonomies of non-functional requirements which already provide lists of quality attributes, e.g. accuracy or reliability [49], so the VBRE method augments analysis of non-functional requirements by drawing attention to issues which might be omitted from a conventional analysis. Most values and motivations held by the stakeholder (self) will be positive, but those related to colleagues, design features and the environment may well be negative, potentially indicating problems that the design needs to solve. Emotional responses of frustration, anxiety and fear during interviews provide corroborating evidence of such problems.

Once the annotation is complete, the transcript is inspected for frequent value and motivation categories, possible causations thereof and differences between individuals and groups. The key issues document may be modified following each cycle of interviews, so it develops incrementally into a rich picture of the stakeholders’ VME.

Finally the implications of the analysis for both the project process and the design are reviewed by referring to the tables and the annotated notes. The output is lists of functional and non-functional requirements, familiar in most RE methods, but also recommendations for organisation, user procedures, functional allocation and work design. More detail on these issues is given in [50].

#### Initial method evaluation

A preliminary evaluation was carried out by applying the method to requirements analysis in two software projects: one investigated geographic information software for public health analysts, while the second concerned project management software for outsourcing. The objective of this evaluation was to assess and improve the usability of the website and then to investigate the perceived relevance of the VME terms for analysts’ practice. The VBRE method was applied in several interviews and insights gained were discussed with colleagues who were also requirements or business analysts, familiar with the same projects. The process of structured, guided reflection was felt to be useful, and indeed directly impacted the projects concerned, in one instance flagging up stakeholders’ different prioritisations about security and audit trails, in the second flagging stakeholders’ anxieties about control and outsourcing which they had been unwilling to express directly.

The main conclusions from this exercise were that the method was useful, but that several concepts in the taxonomy were not easy to understand, so scenarios or narrative examples would help. In particular, more concrete direction and advice would be helpful to novice analysts. More details of this study are given in [34] To make the tables and method process more accessible and usable, a website was developed to explain the tables of VME, with scenarios for elicitation, interview questions and process guidance [51].

### Second version of VBRE

#### Website development

**Fig. 2:**
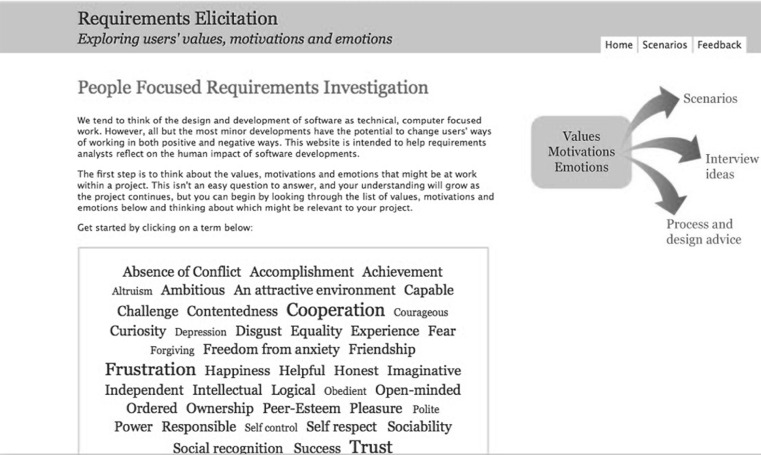
Screenshot of the main VBRE home page

**Fig. 3:**
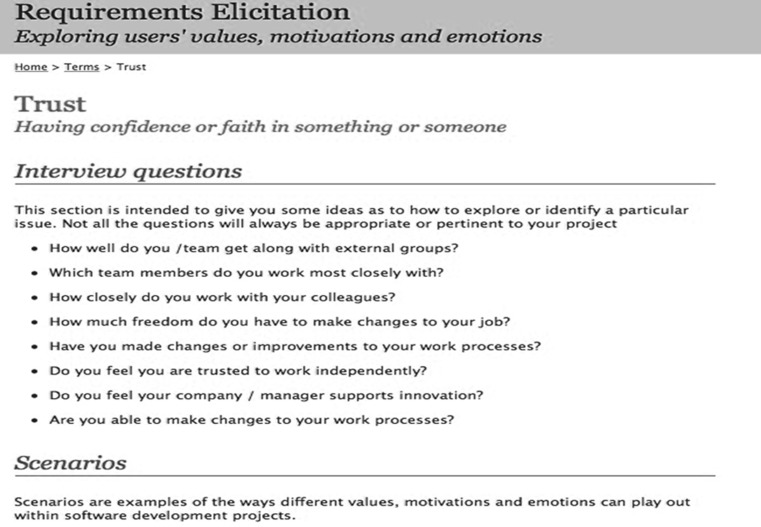
Screenshot of the trust page, listing interview questions, and links to scenarios

The website is structured around the VME tables, with content drawn from the taxonomies (see Fig. 2). This list was collapsed to minimise overlap and ambiguity, emphasising the need for a clear set of prompts. Analysts are encouraged to start exploration of their own project by reviewing this table, and considering which terms might be relevant; a list of prompt questions is provided to help this thought process. Each term has a page of detailed content, including example interview questions, scenarios to help the analyst consider how VME might be important to their project, linked to advice about its potential impact on the requirements process or software design (see Fig. 3 for an example). This content was generated from a number of sources, including project reports, the academic literature and brainstorming sessions with requirements analysts.

**Fig. 4:**
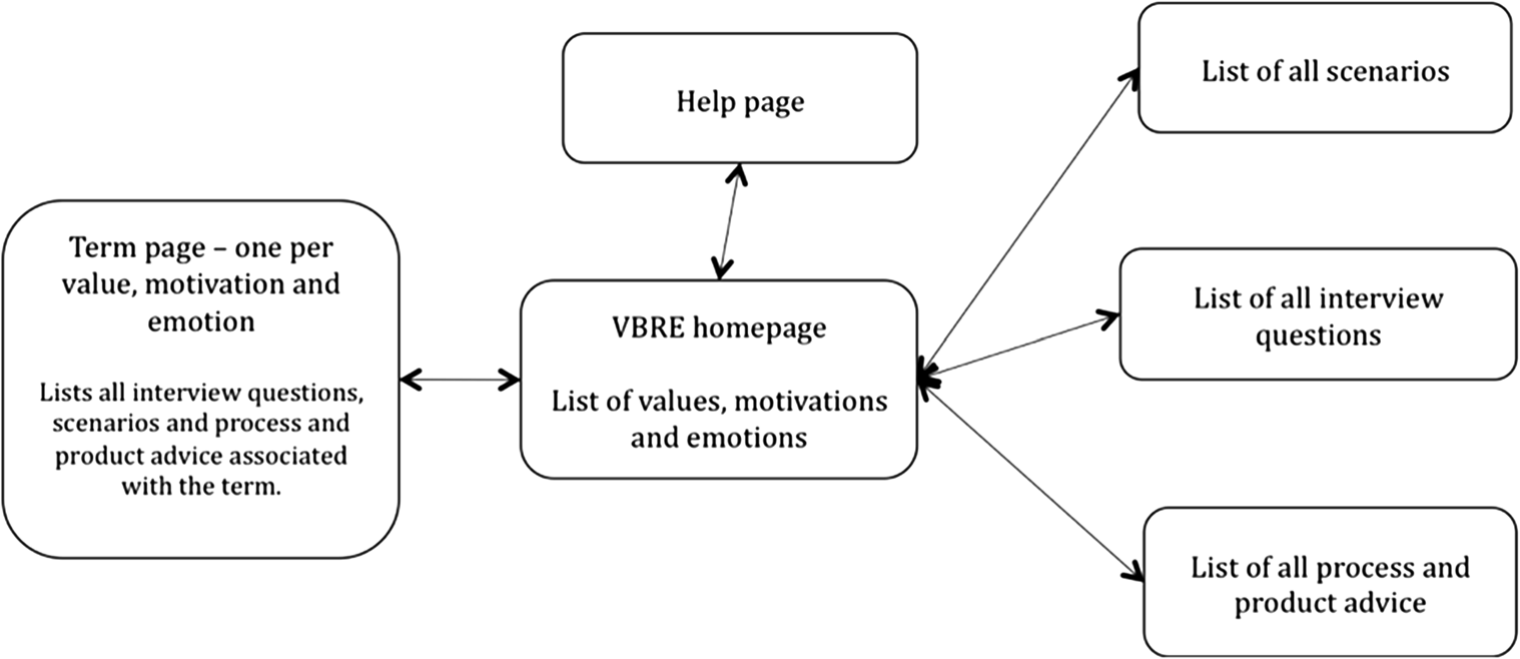
Site map of the VBRE website, showing the main components

The structure of the website is illustrated in Fig. 4. The site contains three main components: scenarios to illustrate all the VMEs, interview questions and process/product advice. Each VME is described in a page with the appropriate scenario, interview question and advice (see Fig. 3), and cross-reference links allow stakeholders to access information either through an index of VMEs or separately through lists of scenarios.

#### Evaluation of the website

An online questionnaire was used to gather feedback on the website. Eighteen respondents were asked to explore the website and then rate its utility and comprehensibility (see Table 4) on a seven-point Likert scale. They were also asked to rate all the VME listed within the website in terms of their relevance to RE problems. The first group of respondents were final year undergraduate students (*n* = 12, 9 male 3 female) who had just completed a course in RE. The students were given an introduction to the website and time to explore the system, before being asked to complete the questionnaire. The second group were professional requirements engineers (*n* = 6, 4 male, 2 female), who were sent an e-mail with a short description of the purpose of the website, and links to the website and the questionnaire. Both groups were asked to analyse values in scenarios using the website.

**Table 4:** Students’ and experts’ rating of the method support website on a seven-point Likert scale

	Students mean (SD)	Experts mean (SD)
Content quality	5.5 (0.85)	6.33 (0.85)
Comprehensibility—contents clear?	6.45 (0.52)	5.66 (0.85)
Comprehensibility—easy to understand?	6.08 (1.03)	4.5 (1.76)
Utility—scenarios	6.17 (0.63)	6.17 (0.98)
Utility—design advice	5.75 (0.75)	4.83 (1.94)
Utility—overall	6.33 (0.84)	6 (1.26)

Both the students and RE experts rated all aspects of the website positively (see Table 4) and felt the site had the potential to be a useful aid to requirements elicitation. Both the students and the analysts felt that the scenarios and associated lists of related VME were very useful. Several students commented that having concrete examples aided their understanding:

They were easy to relate to, you don’t need special knowledge to understand them.

Like the insurance company worried about difficult claims, I don’t know much about insurance but I can understand where they’re coming from, everyone’s dealt with insurance. It was practical.

The experts were slightly more critical of comprehensibility of the concepts and design advice, which is not surprising given their more extensive experience; however, their ratings were well above the mean. Both groups rated each of the VME according to their relevance for requirements analysis. The top ten more relevant terms are illustrated in Table 5. There was agreement between the groups on the relevance of trust, cooperation and frustration.

**Table 5:** Top ten terms relevant to RE rated by students and experts

Students	Mean	Experts	Mean
Trust	6.67	Cooperation	6.83
Cooperation	6.50	Trust	6.83
Achievement	6.50	Frustration	6.83
Frustration	6.50	Absence of conflict	6.50
Success	6.42	Independent	6.50
Absence of conflict	6.33	Ownership	6.50
Happiness	6.33	Equality	6.33
Challenge	6.25	Curiosity	6.33
Experience	6.25	Sociability	6.33
Accomplishment	6.17	Experience	6.17

The experts’ responses were more variable than the students’, which probably reflects diversity in the experts who were drawn from different industries. Hence an analyst working in a creative media or scientific research environment might consider ‘curiosity’ a more important issue than an analyst working in the financial domain who might be more concerned with ‘responsibility’. The students rated all concepts as relevant (mean > 4.0), with the exception of ‘altruism’, which was not understood by eight of the 12 students who asked for an explanation of the term. The expert analysts rated the majority of terms positively, but four terms: ‘forgiving’, ‘depression’, ‘courageous’ and ‘self-control’ were rated at less that 3.5. The popularity of the terms informed the display and link structure in the website.

### Revised (final) VBRE method

The revised method process augmented the description in Sect. 3.1 and is summarised in the following steps:

General advice: Use the emotions table to assess stakeholder reaction to issues raised during interviews. Emotional reactions, particularly to new requirements, may indicate value and motivations which need to be explored. Causes and remedial actions in the Emotions table suggest actions which may be taken in interview and meetings to diffuse adverse affect.

1. Create hunch list of socio-political and VME issues from interviews and other relevant material—observations, scenarios, documentation and workshops.
2. Inspect VME taxonomies and attribute hunches to specific VME categories. Use the implications in the table to structure further analysis with probe questions to explore possible implications. The VBRE website supports this process with scenarios and a link structure to support understanding of VMEs. Attribution may be by explicit mark up using the tables/website for decision support, alternatively the VME tables may be interpreted from memory by experts.
3. Refine hunch lists and VME attribution as analysis progresses. Consider the VME implications of new requirements and plan how these should be presented to stakeholders. Assess the possible emotional impact of value attributed requirements, e.g. anxiety, frustration, arising from suggested changes in working practices.
4. Use the implications and process in the Values and Motivation tables to elaborate requirements, specify non-functional requirements and system architecture design requirements.
5. Continue iterative refinement of understanding stakeholders VMEs. Identify Value and Motivational clashes and organise negotiations to reconcile VME conflicts.

The method consists of the above process advice, the paper-based VME tables and the website support tool. The method is limited to elicitation of VME issues. It does not make any recommendations about how these are translated into design beyond the implications in Tables 1, 2, 3; furthermore, resolution of value conflicts is left to the analysts’ discretion utilising their interpersonal communication and negotiation skills. This version of the method described in this section was applied to all the case studies reported in Sect. 4.

## Case study evaluations and experience

This section reports three evaluations of the VBRE method using case study and action research approaches. The first two evaluations report use of the method first on development of collaborative decision-support tools for epidemiological research, and secondly on development of tools to analyse early onset of dementia from logs of computer use. The third evaluation reports use of the method in industry, supported by the authors in an action research investigation into how the method might be adopted and adapted by others in practice. All three evaluations addressed the research questions about the usability of the VBRE method in practice, the impact of applying the method on requirements outcomes, and how the method was adapted during practice. Since performing a field experiment testing practice with and without the method was impossible given resource limitations, we concentrated on qualitative analysis of method use.

### ADVISES case study

The following experiences emerged during application of the initial (paper-based) method to requirements analysis in the ADVISES project [52]. ADVISES is a decision-support system for academic researchers and National Health Service public health analysts who investigate epidemiology problems. The two distinct stakeholder communities had different goals; for academic researchers, understanding the generic causes of childhood obesity by statistical analysis of health records was a high-level goal. In contrast, the goal of public health analysts was local health management; for example, identifying where best to target interventions, such as promotion of local sports facilities, healthy-eating campaigns. Two academic researchers (both male, age 31, 52) and seven public health analysts (4 male, 3 female, age range 27–41) were recruited through the authors’ personal contacts.

VBRE was introduced in the early stages of project, after terms of references were agreed, through to prototype development. The method was applied by the first author during interviews, scenario-storyboard requirements exploration sessions, and requirements analysis workshops. The stakeholders were encouraged to think aloud and explain their approaches during sessions observing their work practices, which were particularly useful in eliciting values and attitudes to their work. Most meetings and interviews were 60–90 min. Recordings of the various meetings and interviews were transcribed and analysed using the taxonomy and key issues.

The initial list of hunches based on values directly expressed by our stakeholders included being methodical, precise, systematic in their research, while taking a creative approach. The VBRE taxonomies were applied to the hunch list to produce preliminary requirements linked to values and stakeholders’ motivations with cross-references to individual identities and the requirements elicitation documents, e.g. interview transcripts. Requirements and values were refined during further analysis interviews when the requirements and their potential VME implications were discussed with stakeholders. This produced further insights; for example, stakeholders were concerned about their public profile and need to collaborate, though they found sharing of resources difficult, perhaps because of lack of trust.

Following several iterations of VBRE analysis making use of both observation and interview data, we developed a deeper understanding of stakeholders’ values and emotions. Innovation and creativity are key to their research work, but there is a conflict between a strong technical focus and willingness to adopt new software and concerns about control. Collaboration with external groups/people is important for the stakeholders’ research but they rarely share details of their analyses and expressed little trust and confidence in others’ abilities, revealing tension between trust and data security. VMEs elicited by the method informed development of mock-ups and early prototypes, in particular addition of security features to customise data access to particular stakeholder roles. The method also contributed requirements for the socio-technical system design which informed training material and user manuals for data access, and data sharing procedures.

The key issues identified at the beginning of the project were confirmed, and the apparent contradiction between expected and actual external collaboration suggested requirements for better collaborative tools with trust-building measures, e.g. visualisation of work flows and research activities. Security and privacy of data emerged as an important value which was added to the key issues. Sensitivity to the stakeholders’ emotional responses guided the RE process. Stakeholders’ frustrations and anxieties about different aspects of the project were recognised and explored, particularly with the pace of prototype development keeping in step with the requirements elicitation processes. The stakeholders expressed pleasure and engagement when involved in requirements workshops, responding enthusiastically to software prototypes which confirmed the RE approach was appropriate.

**Fig. 5:**
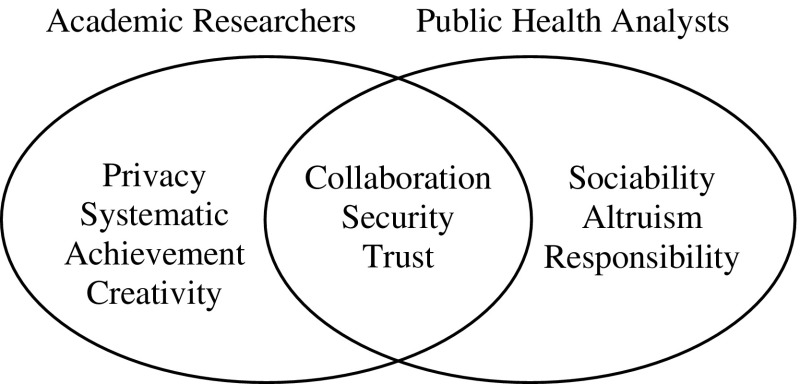
Value map for the two stakeholder groups in the ADVISES project

Values and motivations tables were more useful than the emotions since the system did not provoke any strong emotions, although the stakeholders did express frustration at delays in the prototype evaluation cycles, caused by resource problems. The value map resulting from the analysis is illustrated in Fig. 5.

The academic researchers’ values and motivations emerged from reviewing interview recordings about their work practices. They talked about working in isolation, being responsible for their own success and productivity, the methodical ways they organise and check their work and the care taken to keep their research data and practices secure. Observation of meetings between academic researchers showed them generating and reviewing ideas for new analyses or research. Similarly, interviews and meeting observation provided a rich source of evidence for values and motivations of the public health analysts. They had a strong sense of altruism, both in working with and sharing resources with other analysts, and in a desire to see positive change in the health of children in their local areas.

Collaboration, security and trust were shared values, but there were differences between the stakeholder in design exploration in prototypes, customisation, adaptability and security non-functional requirements. These were addressed by functional requirements for data security on servers, configurable workflows to match systematic or more opportunistic processes, while creative values were supported by interactive visualisation for data analysis. Collaboration and trust were fostered by an iterative user-centred RE process to build up trust within the team, and in the system implementation as a collaborative application. Sociability, altruism and achievement motivations informed decomposition of stakeholder goals. For example achievement, altruism and systematic values led to a subgoal to record analytic procedures, enabling academic researchers to track their own work, while also supporting public health analysts in sharing analysis techniques and results with colleagues.

The major functional requirements (goals) of the systems were for research and analysis support, namely database searches ranging from simple queries to complex associations between variables, leading to display of detailed epidemiological data set in a context with map and graph overviews and functions to compare trends over time and different areas on maps. The VME analysis added further non-functional requirements for privacy and security, while the trust, sociability and collaboration values had implications for CSCW (computer-supported collaborative work) functionality. Systematic, achievement and creativity values for the academic researchers suggested new functional requirements for flexible, extensible analysis tools (creativity support), with a clear process of investigation and audit trail (systematic).

### SAMS case study

The SAMS project aims to increase the proportion of dementia sufferers receiving an early diagnosis by detecting changes in their pattern of computer use. At its core is a set of passive monitors that collect data as the user interacts routinely with their computer. These data are analysed to infer the stakeholders’ cognitive health against a set of clinical indicators representing memory, motor control, use of language, etc. For example, loss of vocabulary is a common symptom of dementia [53]. The VBRE method was applied by the second author during interviews, scenario-storyboard requirements exploration sessions, and requirements analysis workshops. The method was applied from the initial start-up phase of the project, through early requirements analysis with mock-ups and early prototypes to the design phase with fully functional prototypes.

Requirements analysis was initiated with five workshops, conducted with a total of 24 participants (14 male, 10 female, age range 60–75, median 66). Participants were recruited through the Alzheimer’s Society, U3A (University of the Third Age), and local advertising in care homes, GP surgeries and elsewhere. All workshops were structured in two sessions lasting approximately 1 h. In the first session, the SAMS system aims, major components and operation were explained followed by presentation of PowerPoint storyboards illustrating design options for the alert-feedback user interface, such as choice of media (video, text, computer avatars), content (level of detail, social network) and monitoring (periodic feedback, alert-only, explicit tests). The second session focused on discussion of privacy issues in monitoring computer use, data sharing and security, ethical considerations, emotional impact of alert messages, stakeholders’ motivations and likelihood of taking follow-up tests.

Requirements issues raised in the workshops were explored further in 13 interviews presenting scenarios to illustrate similar design options with discussion on privacy, security and ethical issues. Questions in the interviews also probed stakeholders’ reactions to different levels of monitoring (e.g. actions, text) and their perceived trade-off between benefits/motivations versus fears/barriers for adopting the system and taking follow-up action after an alert message. Respondents (4 male, 9 female) ranged in age from 67 to 89 (median 72). The VBRE ‘hunches’ analysis was applied to interview notes and audio recordings of interviews and workshops. The scenarios used in both sessions were designed to test different design approaches that tacitly explored values, such as human-like presence in exploration, social networks (trust, sociability values) and explicit probing issues of security and privacy. The scenarios were designed using the value and motivation taxonomies, e.g. with probe questions to investigate self-efficacy (ability to monitor and control own health), altruism and curiosity. The list of emotions informed analysis of both sessions, with anxiety (invasion of privacy), fear (of diagnosis) and frustration (with computer design and medical professionals) constituting some of the observed reactions.

#### Results: values and emotions

In the workshops, scenarios and prototype designs stimulated only mild emotional reactions with some anxiety over privacy intrusion and accuracy of the computer analysis. No particular design was favoured; however, the avatar and social media options were the least favoured, indicating sociability was not a prominent motivation. All participants expressed concerns over privacy and security arising from monitoring their computer use. Although they were reluctantly willing to share their data with the researchers for analysis, most participants insisted they should have control over their own data. Sharing data with their close kin/friends had to be under their control and the majority would not share information or the alert with their doctor. The majority in all workshops were willing to allow monitoring of their computer use and e-mail text content, made suitably anonymous to protect the identities of other parties to conversations. Most participants expected to experience anxiety and fear if they received an alert message. Contact with a human expert or carer was cited as an important support, with connections to support groups (e.g. the Alzheimer’s Society) for reassurance (empathy) and as additional sources of information to motivate people to take follow-up tests.

As with the workshops, no consensus emerged with the prototypes from the interviews, although more information and sympathetic messages were preferred to the avatar/social media designs, suggesting that self-control (efficacy) was an important value. The respondents were even more concerned about privacy and security, possibly because three participants had recently experienced phishing attacks on the Internet. However, only two individuals were not willing to have their e-mail content monitored. Opinions on minimal data sharing and the need to maintain control over their own data were similar to the workshop participants. The majority of the respondents (11/13) expressed anxiety about being monitored, and expected to experience discomfort, fear and worry when they received an alert message, although ten respondents reported that they could not realistically imagine how they would react in a real-life situation. Five individuals noted that further explanation after the alert message would be vital and all reported that their main motivation for using the system was self-efficacy: a feeling of being in control by self-management of their health.

#### Conclusions: method and requirements implications

Given the diversity of opinion in both the workshops and interviews, prioritising requirements from this analysis was not an easy task. Several values, motivations and possible emotional reactions appeared to have an important bearing on the requirements and design options.

- *Privacy and security values* these were the most common values, with implications for requirements concerning controls over any data sharing, encryption and secure transmission of data to university site, encryption on own PC to mitigate hacking attacks, depersonalised data only for wider research sharing. These values prioritised requirements for secure data transmission, data encryption on client (user) PCs as well as servers, and selective data recording so sensitive data such as home banking and all passwords were not recorded.
- *Trust* in the SAMS system was closely related to the security, but it also involved accuracy of system information and diagnosis as well organisational trust in the universities (system authors), and healthcare professionals. Trust-building requirements included a user control to turn off SAMS recording, and consultation processes for involving users in the research and its results.
- *Motivations* efficacy, desire for self-control, altruism: participation might help research on dementia. Self-control prioritised the user ‘stop recording’ control described above, and for visualisation requirements so users could view summaries of their own recorded activity. Altruism was reflected in publicity material for recruiting participants.
- *Emotion* anxiety and fear of negative alert messages, uncertainty over personal reaction. These emotions led to further analysis of the wording of alert messages, use of graphics and images to explain the rationale behind the message, and options for social configuration, so carers and family members could have access to alerts and their explanation.

The VBRE method helped frame the scenarios and interview questioning strategy, as well as informing the analysis using the taxonomies to review interview notes and hunch lists. VMEs influenced design of data security, including encryption, secure transmission protocols, depersonalisation of data for research purposes, as well as empathetic design of the feedback-alert user interface. Emotional reactions encountered limitations since many participants remarked that they could not anticipate how they would feel when presented in a future reality with emotionally disturbing news (i.e. possible diagnosis of dementia). The scenarios did elicit possible reactions, but doubts were expressed about how accurate this information might be.

### Case study: introducing VBRE into industrial practice

Diary-based case studies were used to validate the effectiveness and utility of the method with practising analysts who used the VBRE method and website in live projects. Diary studies provide a means of studying the effectiveness of the method over time in a real-world setting [54]. The studies aimed to investigate:

- The impact of the method in the projects under study.
- How the method is adapted for use in ‘real life’.

Four requirement analyst volunteers trialled the method in their workplaces. Three were recruited by advertising within local requirements and usability groups. The fourth volunteer was then recruited on the recommendation of one of the existing volunteers who found the method sufficiently useful that he encouraged his colleague to join the evaluation.

#### Evaluation process

An initial interview captured information about analysts’ backgrounds, their past project experience and forthcoming project work. This introductory meeting included a tutorial explaining the VBRE method, the taxonomies and associated guidelines/advice, and how to use the website. The analysts were encouraged to use the method and website as they felt appropriate to their particular project. They kept a record of their experiences of using the method, using a short-structured diary whenever they made use of any component of the method or completed requirements analysis activity on their project. The diary prompts requested information on the RE activity being undertaken, method components used (e.g. hunch list, taxonomies, website), implications (list values, etc., identified), and impact (new and changes to requirements). Interviews were carried out at the end of the study, during which the content of the diary was reviewed. All interviews and meetings were audio-recorded and transcribed.

It was anticipated that the analysts would apply the method to pieces of intermittent requirements work spread over several weeks or months (many of the analysts interviewed worked across several projects at a time, while others carried out multiple roles on their projects, e.g. project manager, user experience or software developer). It was not practical for the researcher to interview the analysts after each application of the method and participants were unlikely to be willing to alert the researcher to every use of the method, therefore diaries were used to collect data about the use of the method. A semi-structured diary format was adopted to facilitate consistent reporting between case studies, while providing some flexibility for participants to document issues or occurrences. Semi-structured approaches are also recommended as they provide participants with some guidance as to how to complete the diary [54]. However, it is inevitable that the degree to which respondents complete the diary will vary and diaries may be incomplete; it is impossible to know how much information has not been reported. Therefore, a final interview reviewed the diary content to clarify any ambiguous entries.

#### Case study results

Table 6 provides an overview of the four studies, describing the requirements elicitation techniques adopted by each analyst, the number of diary entries, components of the method they opted to use and the length of time of the projects.

**Table 6:** Overview of the diary study

Analyst	Requirements elicitation technique	Number of diary entries	Components of VBRE method used	Time period
A1	Interviews (in person)	7	Hunch list VME review Website components: VME list, interview questions, scenarios	4 weeks
A2	Workshops, prototyping	5	Hunch list VME review Website components: VME list, scenarios	7 months
A3	Interviews (in person), telephone conferences	4	Hunch list VME review Website components: VME list	6 months
A4	Workshops	5	Hunch list VME review Website components: VME list, interview questions	3 weeks

Analysts 3 made four formal diary entries but also reported regularly using the front page of the website as an aide memoire before meetings with stakeholders (approximately 1–3 times a week).

The participants were given the freedom to apply the method as they felt most appropriate to their project, and consequently the diary structure was designed to capture information about the ways the method was used, as well as its impact, with the following prompts:

- The frequency of use of the VBRE method
- The circumstances of use: format of the requirements elicitation activity and the situation
- Method components used
- Analyst’s assessment of the impact of the method
- Specific VME
- Problems applying the method.

The analysts kept a record of their experiences of using the method, using a short-structured diary whenever they made use of any component of the method or completed requirements analysis activity on their project. They were also given written guidance on the completion of the diary. Given that each analyst’s work situation was different and that some participants worked across multiple projects, analysts were asked to complete the diary for what they considered to be one phase of their requirements process on one project, covering initial elicitation to requirements definition, rather than to use the method for a set time period. Analysts were asked to contact the researcher to indicate when they felt they had completed an iteration of their requirements work; however, given the length of the evaluations for two of the analysts (6 months, 1 year) the researcher intermittently e-mailed them approximately every eight weeks for progress updates. Interviews were carried out at the end of each study, during which the content of the diary was reviewed and analysts were asked about their perceptions of the utility and usefulness of the method. They were also asked whether they would use the VBRE approach in future. All interviews and meetings were audio-recorded and transcribed.

##### Analyst 1

A1 had a background in nursing and health informatics, but little formal requirements analysis training and had only worked as an analyst on one previous software project, which was badly received by some stakeholders. A1 believed this was due to late and poor stakeholder engagement, and consequently felt anxious about forthcoming stakeholder interviews. He tested the VBRE method in a project developing web-based software to enable the sharing of information about scientific research projects. The initial requirements elicitation consisted of a series of interviews with five research scientists.

###### Method use

A1 followed the VBRE method closely, making an initial hunch list which he updated after reviewing findings from each of his interviews. He used the VBRE website interview questions and scenarios when preparing for interviews. The key VME identified were privacy, ownership, collaboration and altruism.

###### Project impact

When drawing up his preliminary hunch list A1 identified potential issues around the sharing of research data, in particular that researchers did not wish to share information with their rivals, that there might be a lack of interest in collaboration, and that the website might increase speculative contacts requesting data sharing which could be intrusive (privacy, ownership values). He extended his interview plan to include questions to explore whether these concerns were correct. He explored the question of data sharing and developed a more nuanced understanding about what the scientists were and were not willing to share. In contrast to his original expectations, he was surprised to discover that most of the interviewees were happy to share details of their research, viewing it as a way to promote collaboration and an altruistic act which would help novice researchers trying to design their first studies.

A1 also identified a clash in goals between the project funders and the researchers around the sharing of details about biological samples: the funders considered samples collected during projects they had financed to be publicly owned, whereas the researchers were concerned that sharing information might reveal confidential information about their research in advance of publication. This issue was consequently flagged to the project steering committee to develop a policy around sample ownership over the course of a research project. A1 commented that he felt the VBRE method was most useful in helping him prepare for interviews, in particular using the hunch list to prompt him to consider asking questions about stakeholders’ values, while the website interview questions advised on how to broach these subjects.

##### Analyst 2

A2 has a background in bioinformatics and has worked as a requirements analyst for 5 years on a series of large European projects. He tested the VBRE method within one of these projects, developing data management software for an EU consortium. The project faced substantial problems related to trust when it was initiated. Over the course of this case study, some of the original stakeholders left, and new people joined. A2 was concerned about handling this transition and keeping the stakeholder group running smoothly.

###### Method use

A2 used the method to help prepare for the large stakeholder meetings held every 6 months, and then to review the outputs of these meetings. The main components of the method used were the hunch list, the values review, website front page and the scenarios. The key VME identified were openness, tolerance and trust.

###### Project impact

A2 found that drawing up the hunch list and reviewing meeting notes was useful: “a way to tie down things that had been buzzing about in my head for a while” and “it’s a reminder to look at things from a different point of view”. He felt it enabled him to plan the workshops with greater consideration about how to integrate new stakeholders into the existing group. In particular, he highlighted openness, trust and collaboration as positive values that were shared by the existing stakeholders and described wanting to ensure these values were promoted and shared with new members. After the first meeting involving the new members of the group, he identified a need to change how the meetings were run, realising that although the established members were comfortable with each other and used to being direct, and sometimes confrontational, the newer members were quite anxious and shy. To this end, the second workshop was planned with a series of smaller breakout sessions to allow the new and old members to mingle, rather than a large ‘town-hall’ gathering (openness, tolerance, anxiety and trust).

He made some use of the website, commenting that he liked the front page as a reminder of VME issues when planning the analysis sessions and that the scenarios were a valuable way of thinking about the consequences of change. He remarked that “the scenarios helped the interviews go better because I anticipated the issues”.

##### Analyst 3

A3 has a degree in computer science and has worked as a requirements analyst for over 10 years. He is a contractor working in a wide variety of industries and tested the method within a project developing clinical trial software, clinicians, academic researchers and the pharmaceutical industry—he described this project as “technically straightforward but intensely political”. All of the requirements for this project were gathered via workshops and telephone conferences. A3 was confident in running workshops and felt he had good people skills.

###### Method use

A3 started using the VBRE method when he was several months into the project and drew up a hunch list identifying values and emotions covering all the parties involved in the project, which was regularly reviewed and updated in advance of meetings. He made little use of the VBRE website beyond the values list on the homepage. The key VME identified were innovation, ambition, security, and ownership.

###### Project impact

Many of the emotions A3 identified within his project were negative: resistance to change due to concerns about the consequences of failure, anxiety about loss of status. A3 tried to address these concerns and to reinforce positive outcomes of the project. He identified shared, cross-organisation values: an enthusiasm for innovation, as well as ambitions around career progression if the project is a success; speaking to these shared values provided a way to encourage disagreeing stakeholders to find ways to reach consensus on requirements for the sake of the bigger picture.

A3 also made use of his understanding of stakeholders’ values (security, control) in defining system requirements, e.g. building in mechanisms to allow data owners to track and control requests for access to their data, to view reasons for requests and refuse them if considered inappropriate.

A3 felt that the most useful part of the method was drawing up a hunch list and reflecting on the values related to what was happening on the project. He noted that the home page of the website was the most important page for an experienced analyst: “Once I’ve triggered something I can cascade most of what I need but without the front page it slips into the background. There was comparatively little on the website that was new to me, though it was well expressed—I would use it when talking to newer analysts—the main value to me is the reminder list and the intent to leave some time to reflect on this stuff”.

##### Analyst 4

A4 has worked as a commercial software developer for nine years but his computer science degree did not include any RE training, and until his most recent project, he has done little requirements analysis. He is responsible for requirements analysis in support of a tool to collect research data. This involves collecting requirements from nurses and from an external research organisation.

###### Method use

Creation of hunch list, taxonomies, lists of interview questions and scenarios, reflection on stakeholders’ values and, particularly, emotions. The key VME identified were ambition, trust, responsibility and peer-esteem.

###### Project impact

A4 felt the method was helpful in preparing for difficult meetings. He drew up a hunch list and reflected on the different stakeholders’ involvement and felt this enabled him to unpick why the meetings were tense and to think about the participants’ motivations. He identified: ambition, lack of trust, anxiety, extremely high value placed on accuracy, concerns about being perceived as competent and inequality among stakeholders as issues for the project. Beyond this, he was then able to pre-prepare a set of requirements that addressed some of the stakeholder conflicts. A4 commented that he felt more in control of the requirements process and less likely to be surprised. In particular, he realised that key sticking points for getting agreement on the requirements were issues around the nurses’ dependencies on external staff during data collection. A4 did not make use of the scenarios or the design and project advice, commenting that he was very focused on his own project and did not want to think about anything else. He reported feeling better prepared for subsequent meetings due to having anticipated likely problems, and consequently more confident and less anxious.

#### Evaluation summary

All the participants reported that their use of the method had prompted them to consider stakeholders’ VME. All the analysts were able to give examples in which they attributed changes either to their requirements process, or to project requirements as a consequence of applying the method, including:

- Interview and workshop planning
- Challenging analysts’ assumptions about stakeholders’ VMEs
- Reflection on outcomes of meetings
- The addition of new functionality to a project in order to encourage stakeholders to adopt the software.

When asked whether they felt VME were relevant to RE, the analysts primarily focused on stakeholder anxiety, and it is noticeable that anxiety was listed as a relevant VME in three of these case studies; however, a wide range of VME were identified. It is also noticeable that in all the case studies, the analysts identified positive values they wished to promote either within the requirements analysis process, or as qualities of the new system.

The method and website were used extensively in preparing for requirements analysis interviews and workshops. Scenarios, taxonomies and implications guidelines helped anticipate problems, and shaping interview questions to explore problems and find solutions. Less use was made by more experienced analysts of the interview questions and requirements advice, but all found the website provided a useful prompt list to help widen their thinking about social issues and stakeholder values.

Participants were given the freedom to apply the method as they felt appropriate. All drew up hunch lists at the start of their requirements analysis, and felt this was a worthwhile exercise. Both of the novice analysts, A1 and A4, described reviewing some of their interview notes alongside their hunch list. While all the analysts commented on using the website extensively in preparing for meetings, other use of the website varied, with some referring simply to the home page and the taxonomies, while others made use of the detailed guidance. Three of the analysts felt that the full method was time-consuming to apply, although two stated that this would not be a barrier to future use. Analyst A3 had chosen not to apply the ‘cookbook’ version of the method, instead picking and choosing when to visit the website, and had not found this particularly time-consuming. The analysts used a mixture of interviews and workshops as their main requirements elicitation techniques. None reported any issues with integrating the VBRE method into their existing RE approach, understanding the website content or the application of the method.

During the final interview, participants were asked whether they would make use of the method again. Two of the analysts said they would, and A3 commented that he continues to visit the website regularly. A2 does not intend to use VBRE again within his current project but would consider using it when starting a new project. A4 thought that he was unlikely to continue to use the method as it was not part of his company’s standard processes.

Clearly, it is not possible to know whether design decisions and activities undertaken by the analysts are a direct consequence of using VBRE; however, all described using the method to influence their requirements process or understanding of the requirements. For example, A1 raised an issue related to clashing motivations underlying data sharing versus career development, while A2 described changing his approach to workshops to improve the integration of new team members. A3 noted requirements for data access controls, and tracking updates as a consequence of security value concerns, while A4’s analysis of trust values suggested requirements for visibility and checks on data accuracy. A4 felt drawing up the VBRE hunch list enabled him to better understand the causes of conflict within his project, and addressed some of the issues with changes to the system requirements. It is also apparent from the analysts’ hunch lists and diary entries that they paid close attention to their stakeholders’ values, motivations and emotions and acted upon this information.

The generalisability of this evaluation is limited by the small sample size used, and the self-selecting nature of our analyst volunteers. The diary-based approach suffered from the inconsistent reporting at regular intervals and partial conformance to completion instructions; however, it did collect sufficient data from working analysts that allowed us to explore the utility and usability of the method in real life, providing good ecological validity to our evaluation. Gaps in the diary records were followed up in interviews to produce a more complete record of analysts’ activity.

## Discussion and conclusions

### Contributions

The contributions of the VBRE method are first to propose a taxonomy and analysis method to deal with socio-political issues in RE that complement existing analysis of non-functional requirements. The method introduces new considerations into the RE process by drawing attention to individual stakeholders’ VME. Analysis of stakeholders’ values has implications for management of the RE process, as well as providing input to the definition of requirements for personalisation and customisation, extending our previous work on user-centred RE [50, 55]. VBRE surfaces VME issues; however, their resolution in terms of socio-technical design is a matter of negotiation, organisational change and refinement into functional requirements. VBRE provides hints and heuristics towards solutions in Tables 2 and 3, although design implications for system architecture require further research. The method makes no recommendations on handling value clashes and their resolution, although analysis of emotions can give strong indications when such problems exist. To illustrate, in the first case study, VBRE helped to identify conflicting values between the stakeholder and observation of their emotional responses highlighted the issue. However, resolution of the secure access/privacy/openness clash by role access controls was handled by negotiation between the stakeholders, facilitated by the analyst using conventional interpersonal skills.

Although values have received some attention in previous RE approaches, VBRE is the first method to systematically focus on socio-political issues in requirements analysis. Ethnography has been used in several studies to focus on social and political issues in requirements analysis and has aided researchers to understand the socio-technical context in which systems operate, e.g. in the development of air traffic control systems [56], or the use of breast-scanning technology [57]. Ethnography depends on the analyst spending long periods of time in the stakeholders’ workplace, observing and perhaps participating in the work. The approach does not require the analyst to directly ask stakeholders about VME; instead, it relies on the skills and sensitivities of an expert ethnographer to discover values and emotions. Ethnographic techniques are time-consuming to apply, and furthermore there is no guidance for analysts about the identification of stakeholders’ VME. This is in contrast to VBRE which provides a set of taxonomies to prompt and guide the analyst.

Soft systems methodology [18] was designed to support analysts in understanding the wider context in which systems are developed, and highlighted the need for analysts to consider issues such as the likely beneficiaries or losers from the implementation of a system. Although the method does focus on stakeholders’ viewpoint in the Weltanschauung, which may discover values and motivation, the method does not provide any specific guidance about how to identify VME. Value-sensitive design’s main focus is on building values into software, rather than eliciting them from stakeholders [23]. Consequently, the method focuses on a pre-defined set of moral values, using a set of value cards to identify positive values the stakeholders believe the new system should embody. This approach has been used successfully by Friedman [23] and others; however, it limits consideration of VME to a small subset of values with a focus on the system’s socio-political impact. While this focus is useful in exploring more wide ranging ethical and political implications of value (e.g. [58]), it does not give specific guidance about how to interpret values. The extension of value-sensitive design to worth maps [24] adopts a similar hunch list approach to create diagrams of user values and their possible implications; however, this approach does not offer any specific taxonomy of worth or value-related issues. Ethnographers and advocates of participatory design [58, 59] argue that all values can only be understood in a cultural context, we disagree following VSD [23] and believe that providing a priori taxonomy of values is a helpful starting point to guide analysis, although some values will be contextually dependent and change over time, hence we included the open-ended category for socio-political values. Friedman [23] also proposes contextual refinement of values. Ramos and Berry’s [4] guidelines are closely related to the values taxonomy we propose, since they consider structural, social, political and symbolic aspects of the workplace, and the human interactions in the work environment. They not only encourage the analyst to consider information such as processes, regulations and formal job roles but also recommend investigating motivational factors, values and expression of emotions. However, their taxonomies and guidelines are brief and the authors themselves identify the need for more specific advice for analysts. The VBRE method responds to this need by providing detailed VME taxonomies integrated with an analysis process.

### Discussion of results

The development of VBRE was motivated by Mumford’s ETHICS method [17] which provides detailed guidance on identifying values, using a taxonomy and a questionnaire-based approach to support analysts in identifying stakeholders’ values. This method does provide clear guidance to analysts on the identification of stakeholders’ values and emotions but it requires stakeholders to complete a questionnaire about their values, which does not allow more flexible exploration of value-related issues in the interview-based approach we adopted. Practitioners may not find this method acceptable since questionnaires can be time-consuming to complete and need specialist expertise to interpret. VBRE provides specific guidance, and it can be adapted to fit into existing analyst practices, without demanding excessive effort. VBRE also encourages the analyst to consider VME before engaging in elicitation activities such as interviews or workshops, followed by review of the outputs, thereby making use of existing materials.

RE methods for modelling motivation and emotional influences on requirements goals have been proposed [60, 61] following an agent-role, soft-goal modelling approach. However, the People Oriented Software Engineering method [60] did not adopt any specific model of emotion beyond Norman’s framework of three levels of emotional reaction [62], so their role-modelling approach does not provide any specific guidance for analysing the impact of stakeholders’ emotions on requirements. VBRE, in contrast, does provide specific advice based on a sound theory [48]. Emotional requirements could augment modelling of social influences in i* [63], and VBRE could be applied to the goals, skills preferences approach [64] and RE modelling of socio-technical systems. Considering emotions and motivation may help in modelling agents and their relationships, since trust and responsibility are already part of the i* family of models [11, 63]. VBRE could also extend game-based specification methods [65, 66] and requirements for interactive virtual environments such as SecondLife.

Values in VBRE are personal attitudes and beliefs, which we argue, may influence both functional and non-functional requirements. Such personal values contrast with business and monetised concepts of values which have been explored in the *e*-value method [7, 8] and Aurum and Wohlin’s [67] concept of business and product value which is closer to brand reputation and trust in our value taxonomy. Similarly, product value was considered by Hasan et al. [68] in an outline of a method for aligning software releases to business values. Monetised and personal values could be further integrated in RE in trade-offs between the potential monetary benefit in a product and the worth [24] of implementing features that satisfy stakeholders’ personal values.

Research on RE method adoption highlights the need for industry involvement during method development [25]. Testing methods in conjunction with industry partners facilitates identification of issues that might otherwise not have been considered and helps in understanding the practicalities of using a method. Karlsson et al. [69] identified practical issues around scaling up pair-wise evaluation of requirements to meet the needs of an industrial project, while the analysts testing Sommerville et al.’s PREview method [26] liked its flexibility and were content to use the aspects of the method that suited their purposes, disregarding some features entirely. Similarly, incorporating regular feedback from analysts into VBRE method development provided an understanding of how the method would be used in practice from early in the study. Equally, evaluating the VBRE method with industry analysts identified the different ways the novice and expert participants chose to adapt the method to suit their own purposes and circumstances. VBRE was developed by a combination of action research, research-led dissemination of ideas and method practice in industry and participatory design, where development of the method was also a collaborative endeavour. Within the RE community a variety of other approaches have been taken to incorporate industry views into method development. The Volere method was developed by a group of expert RE consultants [1], while methods such as GORE and i* have been refined based on testing involving industry partners [70]. If industry uptake is a marker of success in method development, all these methods can be considered successful; however, these approaches require significant investment of time from individual industry partners.

### Limitations

Threats to validity lie in the small number of case studies which inevitably limits generalisation of our results; however, the evaluation did cover several projects and different domains in industry and medical decision-support systems (ADVISES, SAMS), which provides some confidence in application of the VBRE method. While semicontrolled field experiments comparing VBRE and a control condition might have produced validation evidence, it is questionable whether such evidence would have any external validity since controlling the context beyond experiments using student assignments are impractical in industrial settings; consequently the case study approach provides better ecological validity. Case study findings were based on triangulation of evidence, although other, more wide ranging quantitative methods (e.g. surveys) could have supplemented the findings. We preferred to focus on qualitative research to yield insight into how the method was used and adapted at the expense of wider ranging investigation into the possible acceptability of the method which could only be realised by evaluation of a large scale tutorial programme. Basing the method taxonomies on accepted sources in the literature provides construct validity, which was supplemented by card-sort testing of values in the website evaluation. VBRE is applicable to eliciting values, motivations and emotions from a wide variety of stakeholder roles, e.g. operator end users, managers, system owner as well as designers. Validation has focussed on users who will become system operators and managers, with requirements engineers. Further validation and adaptation of the method will be necessary to tailor it to secondary stakeholders, e.g. strategic level managers and stakeholder consumers in market orient RE.

### Future directions and conclusions

In future work, we will extend the evaluations we have carried out so far and evaluate method usability and effectiveness after a tutorial programme, although we believe there is no substitute for evaluation of actual practice in industry, our ongoing aim. Other future directions are to integrate values more closely into RE methods by mappings to non-functional requirements [71]. The framework advances previous elicitation techniques by providing explicit taxonomies of values and motivations to guide discovery of socio-political issues. It could extend the goals–skills preference trade-offs [64] by providing a sound taxonomy of personal characteristics and motivations. While our approach so far has been inspection based, a possible extension could be to use elements of the taxonomy in modelling soft-goal analysis in the i* framework [11] and augmenting the semantics of i* agent properties and relationships.

One disadvantage of taxonomic approaches is that they become cumbersome to use, and we have anticipated this by providing a website to support use of the method. This gives practical, tangible support around the elicitation and exploration of stakeholder values, motivations and emotions. The taxonomic approach in VBRE could be profitably combined with Value-Sensitive Design [23, 72] to integrate our value categories with VSD’s image cues and scenario based approach to stimulate consideration of value oriented design implications. Resolution of VME implications in all RE approaches will require collaborative conversations between stakeholders to discover possible design solutions, their socio-political implications and then agree a consensus. This process could be supported by linking values more closely with software architecture, thereby enabling requirements engineers and stakeholders to evaluate design trade-offs. A start in this direction has been made by considering how values such as creativity may be supported by software components to customise and compose systems from components, while trust values may have implications for components that promote transparency, e.g. visualisation of each person’s activity in collaborative systems [73].

The VBRE framework accommodates novice and expert practice, by describing different pathways for the method knowledge to be used directly as aide memoires or learned and used directly. This flexibility was well received in the initial industrial trials. Although experience and validation results for the method are promising, the data are preliminary. Future work will focus on further evaluation with industrial practitioners.
